# Sexual Orientation Disparity in Suicidality: The Indirect Effects of Perceived Pressure to Get Married, Perceived Burdensomeness, and Thwarted Belongingness among Unmarried Chinese Adults

**DOI:** 10.1007/s10508-025-03159-6

**Published:** 2025-06-04

**Authors:** Fangsong Liu, Eddie S. K. Chong, Qiyao Jiang

**Affiliations:** 1 School of Psychology, Jiangxi Normal University, Nanchang, China; 2 Center of Mental Health Education and Research, Jiangxi Normal University, Nanchang, China; 3 Department of Social Work and Social Administration, The University of Hong Kong, Room 520, The Jockey Club Tower, Pokfulam, Hong Kong; 4 International Department, Shenzhen Middle School, Shenzhen, China

**Keywords:** Sexual orientation, Perceived burdensomeness, Thwarted belongingness, Suicidality, Marriage, Mainland China

## Abstract

The present study examined the indirect relationship between sexual orientation status and suicidality through perceived parental, social, and internalized pressure to get married, as well as perceived burdensomeness and thwarted belongingness. An online sample of 1420 Chinese unmarried adults (54.4% female, 41.8% male, and 3.9% non-binary; age: *M* = 25.4 years, *SD* = 4.8) completed self-report measures. The results showed that sexual minority individuals reported higher levels of perceived parental pressure to get married, social pressure to get married, perceived burdensomeness, thwarted belongingness, and suicidality, and lower levels of internalized parental pressure to get married compared to their heterosexual counterparts. Path analyses suggested that being a sexual minority was associated with increased suicidality through perceived burdensomeness and thwarted belongingness, as well as through social pressure to get married and then thwarted belongingness. Furthermore, being a sexual minority was indirectly associated with increased suicidality through internalized pressure to get married. These findings contribute to our understanding of how sexual orientation status contributes to suicidality among Chinese unmarried adults.

## Introduction

Sexual minority individuals often experience higher rates of suicidal ideation and behaviors than their heterosexual counterparts (Marshal et al., 2011). The minority stress theory posits that sexual minority stress contributes to this elevated risk (Meyer, 2003), which is supported by substantial empirical evidence (de Lange et al., 2022; Fulginiti et al., 2020). Among Chinese sexual minorities who face cultural, societal, and familial expectations to establish heterosexual marriages, perceived parental, social, and internalized pressure to get married has begun to receive attention in mental health research (Zheng et al., 2020). Establishing heterosexual marriage and raising children in a heterosexual family are deeply ingrained social norms in Mainland China (Li et al., 2015a). A qualitative study found that unmarried adults in China face tremendous external pressure to get married and experience a sense of guilt and shame for not fulfilling such expectations (Gui, 2023). Due to the prevalence of heteronormativity and the lack of public recognition of same-sex relationships in China, sexual minorities may be more likely to experience such pressures than heterosexual individuals. Zheng et al. (2020) found a positive association between perceived external pressure to get married and psychological distress within a sexual minority sample. However, this study did not examine the disparity in perceived parental, social, and internalized pressure to get married across different sexual orientations, nor their psychological consequences, including suicidal ideation, attempts, and behaviors.

The interpersonal theory of suicide proposes that perceived burdensomeness and thwarted belongingness are key factors leading to suicidality (Van Orden et al., 2010). Perceived burdensomeness refers to the persistent belief that one is a burden to others, reflecting a distorted perception that one’s death is more valuable than one’s life. In contrast, thwarted belongingness refers to a negative cognitive-affective state characterized by feelings of loneliness and the absence of reciprocal caring relationships. Previous studies have shown that life stressors contribute to suicidality through these factors (Bhargav & Swords, 2022; Gratz et al., 2020). As salient stressors for unmarried adults, perceived parental, social, and internalized pressure to get married may also correlate with suicidality via perceived burdensomeness and thwarted belongingness. Therefore, we investigated whether sexual orientation status would be indirectly linked with suicidality through these aspects of marriage-related pressure and then perceived burdensomeness and thwarted belongingness.

### Three Types of Perceived Pressure to Get Married

In contemporary China, marriage and family are considered as the social norms, leading to substantial pressure on unmarried adults to get married (Gui, 2023). Filial piety, a key aspect of Confucianism, requires an individual to fulfill familial roles, including engaging in a heterosexual partnership to maintain the family lineage (Bedford & Yeh, 2021). If individuals do not marry by what is considered an appropriate age, some parents may believe their adult children have not fulfilled their familial responsibilities and may urge them to get married. Additionally, in response to a slowdown of economic growth and low marriage and birth rates, the government has abolished the one-child policy, encouraging couples to have two children (Zeng & Hesketh, 2016). Moreover, various social figures, including siblings, relatives, friends, colleagues, and supervisors, may express concern regarding one’s unmarried status (Zheng et al., 2020). As a result, unmarried adults may perceive their singlehood as a failure to meet social, cultural, and familial expectations, leading to increased stress (Zhu et al., 2022). Furthermore, due to the prevailing norms surrounding heterosexual marriage and procreation, individuals may internalize these societal and cultural expectations, believing that marriage is a crucial rite of passage into adulthood (Zheng et al., 2020). Therefore, Zheng et al. (2020) proposed that the pressure to get married can be categorized into three types: parental pressure, social pressure, and internalized pressure.

In a heteronormative environment, heterosexual individuals who experience pressure to get married could establish heterosexual marriages that align with their sexual orientation. Such pressure is likely to diminish after heterosexual individuals develop romantic relationships and get married. In contrast, the lack of formal recognition for same-sex relationships may complicate matters for sexual minorities, making it more challenging to navigate similar pressures to get married. Research suggests that Chinese sexual minorities may enter heterosexual marriages to meet parental and social expectations. For example, interviews with Chinese sexual minorities revealed that many expressed intentions to engage in “marriage fraud” (i.e., marrying a heterosexual person who is unaware of their sexual orientation) or a “formality marriage” (i.e., marrying a different-sex partner who also identifies as a sexual minority) (Ren et al., 2019). This suggests that the pressure to conform to the expectations of marriage becomes so strong that sexual minorities may opt for heterosexual unions, even when these arrangements do not affirm their sexual orientation. In addition, because sexual minority individuals can experience emotional or sexual attraction to people of the same sex, they may be less inclined to identify with sociocultural norms that expect one to marry someone of a different sex. Therefore, we hypothesized that unmarried sexual minority adults would experience higher levels of perceived parental and social pressure to get married, but lower levels of internalized pressure to get married compared to their heterosexual counterparts.

A limited body of empirical research has examined the effect of perceived pressure to get married on psychological distress. Zheng et al. (2020), for instance, revealed a robust association between external pressure to get married—particularly from parents and broader societal norms—and psychological distress among sexual minority adults in China. In contrast, only a weak association emerged between internalized marital pressure and psychological distress. However, quantitative studies exploring the relationship between the three types of perceived marital pressure and suicidality remain sparse. In addition, a qualitative study conducted by Pridmore and Walter (2013) revealed that forced marriages could lead to suicidality, suggesting that external pressure to enter a heterosexual marriage without genuine intent to cultivate such a relationship may serve as a risk factor for suicidality. Research among rural young adults found that the typical negative life events preceding suicidality included stressors related to family relations, love affairs, and marital issues (Zhang & Ma, 2012). This observed link between external pressures to get married and suicidality has also been corroborated by news report (Xu, 2016). Therefore, we hypothesized that perceived parental and social pressure to get married would be positively associated with suicidality. Given the limited insights from previous studies regarding the effect of internalized pressure to get married on suicidality, we refrained from making a definite hypothesis about this relationship.

### Perceived Burdensomeness and Thwarted Belongingness

The interpersonal theory of suicide has been widely used to account for the occurrence of suicidality (Chu et al., 2017; Van Orden et al., 2010). Due to heteronormative beliefs and sexual orientation-based discrimination, sexual minority individuals often experience heightened levels of perceived burdensomeness and thwarted belongingness, which in turn elevate their risk of suicidality (Baams et al., 2015). While past research has provided empirical evidence supporting the significant impact of perceived burdensomeness and thwarted belongingness on suicidality among the sexual minority population, perceived burdensomeness has been found to be a more consistent predictor when compared to thwarted belongingness (Chu et al., 2017; Moody et al., 2023). Of note, most studies using this framework have predominantly focused on Western cultures. Given that Chinese culture often places greater value on interpersonal relationships than on individualism (Markus & Kitayama, 2010), it remains unclear whether perceived burdensomeness exerts a stronger effect on suicidality than thwarted belongingness within this cultural context.

Two studies conducted in Western contexts have found that sexual minority college students endorsed higher levels of perceived burdensomeness than their heterosexual counterparts (Hill & Pettit, 2012; Pate & Anestis, 2020). In addition, it has been argued that sexual minorities may conceal their sexual orientation due to acceptance concerns (Meyer, 2003), which can disrupt their emotional connections with significant others and increase feelings of loneliness. However, researchers found no empirical evidence indicating elevated levels of thwarted belongingness among sexual minority colleges students in the U.S. as compared to their heterosexual peers (Hill & Pettit, 2012; Pate & Anestis, 2020). One possible explanation for the similar levels of thwarted belongingness observed in these studies is that sexual minority individuals in universities often have greater opportunities to form close friendships within or outside the sexual minority community for support regarding their sexual orientation, thus buffering their feelings of thwarted belongingness (Pate & Anestis, 2020). While attitudes towards sexual minority people keep evolving in contemporary China, stigma targeting sexual minority orientations still warrants attention (Suen et al., 2022). Within this context, some sexual minority individuals may experience guilt and self-blame, believing that they are burdensome to others, such as their family members. They may also find it more challenging to develop connections and a sense of belongingness that would allow them to discuss marriage-related concerns, compared to their heterosexual peers. Hence, we hypothesized that unmarried sexual minority adults in China would report higher levels of perceived burdensomeness and thwarted belongingness than their heterosexual counterparts.

The literature frequently examines the indirect effects of perceived burdensomeness and thwarted belongingness on the negative impact of stressful life events on suicidality across various population (Bhargav & Swords, 2022; Gratz et al., 2020). Although pressure to get married could be a significant stressor for unmarried adults in China, its associations with perceived burdensomeness and thwarted belongingness have not been examined. Sharp and Ganong (2011) found that single women felt unsuccessful due to societal reactions regarding their unmarried status, implying that pressure to get married may lead unmarried adults to perceive themselves as failures in the eyes of their parents and society at large, thereby increasing their sense of perceived burdensomeness. Moreover, it has been noted that Chinese sexual minority individuals often psychologically or physically disconnect themselves from family members as a means of coping with family pressure to get married (Chang, 2015; Liu, 2019; Lo, 2020), suggesting that external pressures related to marriage may positively correlate with feelings of thwarted belongingness.

### The Present Study

**Fig. 1:**
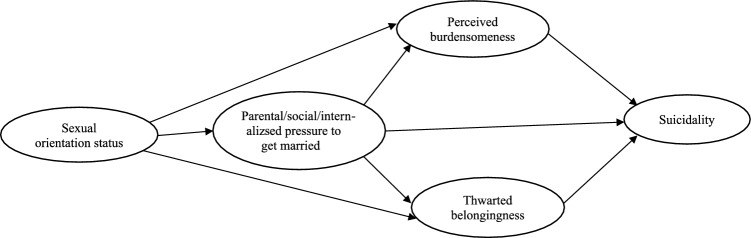
Research model

Based on the aforementioned theories and empirical evidence, we proposed the following hypotheses: (1) Unmarried sexual minority adults would have higher levels of perceived parental and social pressure to get married, perceived burdensomeness, thwarted belongingness, and suicidality, and lower levels of internalized pressure to get married than their heterosexual peers, (2) sexual orientation status would have an indirect association with suicidality through perceived parental and social pressure to get married and then perceived burdensomeness and thwarted belongingness; specifically, being a sexual minority would be associated with higher levels of perceived parental and social pressure to get married, which in turn would be associated with higher levels of perceived burdensomeness and thwarted belongingness, which would subsequently contribute to higher levels of suicidality. In addition, we raised a research question: Does sexual orientation status have a two-step indirect association with suicidality through internalized pressure and then through perceived burdensomeness and thwarted belongingness? The hypothesized indirect path model is illustrated in Fig. 1.

## Method

### Participants and Procedure

This study was part of a broader research program that received ethical approval from Jiangxi Normal University (IRB Number: JXNU-PSY-2024028) and the University of Hong Kong (IRB Number: EA220034) and adhered to APA ethical standards regarding participant treatment. From March to May 2022, the first author reached out to LGBT+ centers to recruit sexual minority individuals. These centers supported the dissemination of recruitment advertisement through their WeChat Official Accounts, subscribed by many Chinese sexual minorities. Concurrently, all authors shared the recruitment advertisement which consisted of research aims and survey links to personal WeChat groups and friend circle, encouraging recipients to further distribute the advertisement among their contacts. Interested participants could access the online survey by clicking on the provided links or scanning the QR code, which led them to a consent form on the first page. The consent form outlined the confidentiality of their data, their right to withdraw from the survey at any time without any cost, and potential benefits for completing this survey. Participants were required to select “Yes, I agree to participate in this study” before proceeding to the survey items. The average time taken to complete the survey items was 875 s. Participants who completed the survey received a compensation of 6 RMB for their time and efforts. To test the hypothesized path model, relevant variable data were extracted from the completed surveys for subsequent analyses.

A total of 1758 participants completed the online survey. Based on the inclusion criteria for participants who were at least 18 years old, living in China, and unmarried, 180 participants were excluded due to being under 18 years of age or married. In addition, 158 participants were excluded through three methods designed to identify careless responses: they either provided an incorrect answer to a specific validity item (i.e., please choose the second option for this item; Liu et al., 2023b), responded to each item in less than 2 s (Huang et al., 2012), or self-reported that their data were not authentic and should not be included in subsequent analyses in a specific item at the end of the survey (Meade & Craig, 2012). In the end, 1420 participants (54.44% female, 41.76% male, and 3.8% non-binary; age: *M* = 25.4, *SD* = 4.8) were retained for subsequent data analyses. The specific demographic information was illustrated in Table 1. Compared with demographic information (i.e., gender, age, education, and place of birth) of the general population from the seventh national census in 2020 (National Bureau of Statistics, 2021), the respondents in the present sample exhibited a higher proportion of females, were younger, possessed higher levels of education, and were more likely to be born in urban areas. Notably, over 45.3% of participants in the presents study reported personal monthly income exceeding the median monthly income of approximately 2753 RMB (around US$387) derived from the Chinese population in 2023 (National Bureau of Statistics, 2024).

**Table 1:** Demographic information of the participants and their scale scores (N = 1420)

Demographic variables	N (%)	PPGM (M/SD)	IPGM (M/SD)	SPGM (M/SD)	PB (M/SD)	TB (M/SD)	Suicidality (M/SD)
Gender							
Male	593 (41.76%)	3.30 (1.05)	2.55 (0.94)	2.97 (1.07)	2.08 (1.38)	2.95 (1.20)	1.67 (0.89)
Female	773 (54.44%)	3.00 (0.98)	2.29 (0.87)	2.57 (1.01)	1.83 (1.16)	2.69 (1.17)	1.65 (0.86)
Non-binary	54 (3.80%)	2.92 (0.84)	2.53 (0.95)	2.56 (0.72)	2.86 (1.73)	3.59 (1.13)	2.48 (1.10)
Sexual orientation							
Heterosexual	512 (36.06%)	3.00 (0.97)	2.44 (0.85)	2.63 (1.02))	2.48 (1.04)	2.48 (1.04)	1.35 (0.67)
Gay/lesbian	625 (44.01%)	3.27 (1.05)	2.41 (0.94)	2.89 (1.07)	3.04 (1.25)	3.04 (1.25)	1.84 (0.94)
Bisexual	183 (12.89%)	3.13 (0.91)	2.43 (0.93)	2.74 (0.99)	2.88 (1.18)	2.88 (1.18)	1.82 (0.87)
Pansexual	44 (3.10%)	2.81 (1.10)	2.00 (0.84))	2.23 (1.58))	3.12 (1.17)	3.12 (1.17)	2.07 (0.89)
Asexual	21 (1.48%)	2.45 (0.95)	2.05 (1.00)	3.21 (1.83)	3.17 (1.28)	3.17 (1.28)	2.93 (1.28))
Questioning	17 (1.20%)	3.21 (0.89)	2.84 (1.03)	2.46 (1.25)	3.25 (1.13)	3.25 (1.13)	1.87 (0.96)
Queer	9 (0.63%)	2.61 (1.11)	2.50 (1.16)	1.81 (1.25)	3.35 (1.53)	3.35 (1.53)	2.42 (1.12)
Others (unspecified)	9 (0.63%)	2.25 (1.03)	2.53 (0.96)	3.46 (1.68)	3.96 (0.76)	3.96 (0.76)	2.11 (0.83)
Place of birth							
Urban area	982 (69.15%)	3.03 (1.02)	2.34 (0.90)	2.66 (1.04)	1.97 (1.29)	2.82 (1.21)	1.72 (0.89)
Rural area	438 (30.85%)	3.32 (0.98)	2.55 (0.92)	2.91 (1.04)	1.98 (1.32)	2.87 (1.18)	1.64 (0.89)
Educational attainment							
High school or below	134 (9.44%)	3.00 (1.00)	2.54 (0.94)	2.75 (1.01)	2.50 (1.61)	3.13 (1.27)	1.96 (1.00)
Associate degree	100 (7.04%)	3.29 (0.90)	2.54 (0.94)	2.86 (1.09)	2.51 (1.63)	3.12 (1.29)	1.99 (0.98)
Bachelor's degree	718 (50.56%)	3.17 (1.00)	2.37 (0.92)	2.78 (1.05)	2.02 (1.31)	2.90 (1.19)	1.75 (0.91)
Master's degree	406 (28.56%)	3.07 (1.05)	2.42 (0.90)	2.69 (1.05)	1.69 (1.02)	2.67 (1.16)	1.50 (0.78)
Doctoral degree	62 (4.37%)	2.85 (1.00)	2.29 (0.77)	2.35 (0.87)	1.27 (0.51)	2.08 (0.73)	1.21 (0.63)
Monthly income							
Less than 2000 RMB (286 USD)	533 (37.54%)	3.05 (1.00)	2.39 (0.89)	2.76 (1.00)	2.07 (1.35)	2.85 (1.18)	1.76 (0.92)
2000–4999 RMB (714 USD)	244 (17.18%)	3.17 (0.98)	2.57 (0.96)	2.82 (1.09)	2.14 (1.32)	3.00 (1.25)	1.74 (0.92)
5000–9999 RMB (1428 USD)	315 (22.18%)	3.18 (1.00)	2.33 (0.88)	2.72 (1.01)	1.95 (1.31)	2.84 (1.21)	1.75 (0.90)
10,000–19,999 RMB (2856 USD)	241 (16.97%)	3.24 (1.00)	2.41 (0.93)	2.75 (1.08)	1.79 (1.19)	2.77 (1.17)	1.55 (0.82)
More than 20,000 RMB	87 (6.13%)	2.82 (1.19)	2.37 (0.96)	2.38 (1.12)	1.46 (1.02)	2.37 (1.10)	1.31 (0.70)

PPGM = Parental pressure to get married. SPGM = Social pressure to get married. IPGM = Internalized pressure to get married. PB = Perceived burdensomeness. TB = Thwarted belongingness. M = Mean. SD = Standard deviation

### Measures

#### Demographic Variables

Participants’ demographic information including age, gender, sexual orientation, place of birth, educational attainment, and monthly income were collected.

#### Perceived Pressure to Get Married

The Perceived Pressure to Get Married Scale (Zheng et al., 2020) is an 11-item scale designed to measure the degree to which Chinese sexual minorities perceive pressure to get married. This scale is consisted of three subscales (i.e., Perceived Parental, Social, and Internalized Pressure to Get Married). Respondents reported the extent to which they agreed with items on a five-point Likert-type scale, ranging from 1 (*strongly disagree*) to 5 (*strongly agree*). This scale has demonstrated acceptable internal reliability and validity among Chinese sexual minorities (Liu et al., 2023a; Zheng et al., 2020). For example, both exploratory factor analysis and confirmatory factor analysis (CFA) provided evidence supporting a three-factor model among Chinese sexual minorities (Zheng et al., 2020). Additionally, the internal reliability coefficients for social pressure, parental pressure, and internalized pressure were 0.82, 0.88, and 0.77, respectively (Zheng et al., 2020). Sample items for the Perceived Parental, Social, Internalized Pressure to Get Married subscales were “I feel stressed because my parents often pressure me to get married,” “I am always worried that others may talk about my marital status,” and “One should get married at the appropriate age,” respectively.

Given that this scale had not been applied to heterosexual individuals, CFA was conducted to assess the construct validity of a three-factor model among heterosexual participants. The findings demonstrated that this model exhibited a satisfactory fit to the data among heterosexual participants (CFI = 0.956; TLI = 0.935; SRMR = 0.054; RMSEA = 0.075, 90% CI [0.063, 0.089]). Additionally, the model also fit the data for sexual minority participants (CFI = 0.961; TLI = 0.942; SRMR = 0.035; RMSEA = 0.073, 90% CI [0.063, 0.082]) and across all participants (CFI = 0.960; TLI = 0.940; SRMR = 0.043; RMSEA = 0.073, 90% CI [0.066, 0.081]). Furthermore, based on the present sample, the Cronbach’s *α* values for the Perceived Parental, Social, and Internalized Pressure to Get Married subscales were 0.85, 0.85, and 0.72 for all participants; 0.82, 0.85, and 0.73 for heterosexual participants; and 0.86, 0.85, and 0.69 for sexual minority participants. These results supported the validity and reliability of this measure among both heterosexual and sexual minority individuals. The mean scores of the three subscales were used in subsequent data analyses and higher scores indicated higher levels of perceived parental, social, and internalized pressure to get married.

#### Perceived Burdensomeness and Thwarted Belongingness

The 15-item Interpersonal Needs Questionnaire (INQ; Van Orden et al., 2010) was used to assess the extent to which sexual minorities have unmet interpersonal needs regarding self-worth and sense of belongingness. Li et al. (2015b) has translated and validated the Chinese version of INQ, which was used in the present study. This scale includes Perceived Burdensomeness (6 items) and Thwarted Belongingness (9 items) subscales. Participants responded on a 7-point Likert format, ranging from 1 (*Not at all true for me*) to 7 (*Very true for me*). Sample items for the Perceived Burdensomeness and Thwarted Belongingness subscales were “I think I am a burden on society” and “I think my death would be a relief to the people in my life,” respectively. Previous studies have shown that Perceived Burdensomeness and Thwarted Belongingness subscales exhibited adequate internal consistency reliability among Chinese sexual minorities (Liu et al., 2023b) as well as sexual minority youths in the Netherlands (Baams et al., 2015). Furthermore, both perceived burdensomeness and thwarted belongingness demonstrated convergent associations with related interpersonal constructs, thereby supporting their construct validity (Van Orden et al., 2012). Based on the present sample, CFA was conducted to assess the construct validity of a two-factor model among all participants, indicating that this model exhibited a satisfactory fit to the data (CFI = 0.957; TLI = 0.947; SRMR = 0.075; RMSEA = 0.077, 90% CI [0.072, 0.082]). The Cronbach’s* α* values in the presents study for the Perceived Burdensomeness and Thwarted Belongingness subscales were 0.95 and 0.90, respectively. Higher Perceived Burdensomeness and Thwarted Belongingness subscale scores indicated higher levels of perceived burdensomeness and thwarted belongingness.

#### Suicidality

The four-item Suicidal Behaviors Questionnaire-Revised (SBQ-R; Osman et al., 2001) was used to measure participants’ suicidality (i.e., ideation, attempt, and behavior). Shi et al. (2021) has translated and validated this scale among Chinese college students, which was used in the present study. Previous studies supported that the SBQ-R is an effective and reliable tool for suicide assessment in clinical and non-clinical samples (Osman et al., 2001; Shi et al., 2021). For example, among Chinese college students, the SBQ-R exhibited significant positive correlations with the nine-item Patient Health Questionnaire and the seven-item Generalized Anxiety Disorder scale (Shi et al., 2021). The internal reliability, split-half reliability, and test–retest reliability coefficients for this scale were 0.75, 0.78, and 0.62, respectively (Shi et al., 2021). A sample item was “Have you ever thought about or attempted to kill yourself?” CFA was conducted to assess the construct validity of a one-factor model among all participants, indicating that this model exhibited a satisfactory fit to the data (CFI = 1; TLI = 1; SRMR = 0.002; RMSEA = 0.000, 90% CI [0.000, 0.063]). In the present sample, the Cronbach’s *α* value of this scale was 0.80. A higher scale score indicated a higher level of suicidality.

### Statistical Analyses

SPSS 22 was used to perform descriptive analyses. The skewness and kurtosis values of the variables in the hypothesized model suggested that these variables conformed to normal distribution. Moreover, the Mahalanobis distance test indicated none of multivariate outliers. Pearson correlation coefficients between continuous variables were calculated for bivariate correlations. Multivariate analysis of variance (MANOVA) was conducted to investigate whether there would be difference in suicidality within categorical demographic variables. If there were significant difference in suicidality within demographic variables, they would be included as covariates in subsequent analyses.

Structural equation modeling was conducted using Mplus 7.4 to examine the proposed indirect model. We firstly examined the measurement model using CFA to see whether the observed variables could adequately assess their corresponding latent variables. Subsequently, the structural model was examined to investigate the indirect association between sexual orientation status and suicidality through three types of perceived pressure to get married and then perceived burdensomeness and thwarted belongingness among the overall participant sample. Additionally, we further investigated this structural model using two subsamples: one comparing lesbian/gay and heterosexual individuals, and the other comparing bisexual and heterosexual individuals. However, given that the sample sizes of asexual, pansexual, questioning, queer, and other sexual minority groups were small, we did not examine the structural model within subsamples comprising the above-mentioned sexual minority and heterosexual individuals. The fit of the measurement and structural models was determined by the following indices: Tucker-Lewis index (TLI) ≥ 0.9, comparative fit index (CFI) ≥ 0.9, root mean square error of approximation (RMSEA) ≤ 0.08, and standardized root mean square residual (SRMR) ≤ 0.08 (Hu & Bentler, 1999). To obtain a more precise standard error estimate, we used a bootstrap procedure (1,000 bootstrap samples) to estimate 95% bias-corrected bootstrap confidence intervals (CI) in the indirect analysis. The indirect effect was significant when the 95% CI did not include zero.

## Results

### Descriptive Analysis

Table 2 shows the means and standard deviations of all continuous variables, and bivariate correlations among variables. Perceived parental, social, and internalized pressure to get married were positively correlated with perceived burdensomeness and thwarted belongingness. Moreover, perceived parental and social pressure to get married were positively associated with suicidality. In addition, perceived burdensomeness and thwarted belongingness had positive correlations with suicidality. In addition, being younger was correlated with higher levels of suicidality.

**Table 2:** Means, SDs, and correlational coefficients for variables

Variables	1	2	3	4	5	6	7
1. Age	1.00						
2. Parental pressure	.05^*^	1.00					
3. Social pressure	.02	.66^**^	1.00				
4. Internalized pressure	.02	.31^**^	.46^**^	1.00			
5. PB	− .11^**^	.18^**^	.19^**^	.14^**^	1.00		
6. TB	− .06^*^	.17^**^	.21^**^	.15^**^	.56^**^	1.00	
7. Suicidality	− .13^**^	.09^**^	.08^**^	.03	.54^**^	.45^**^	1.00
*M*	25.43	3.12	2.74	2.41	1.97	2.83	1.69
*SD*	4.80	1.01	1.04	0.91	1.30	1.20	0.89

PB = Perceived burdensomeness. TB = Thwarted belongingness

M = Mean. SD = Standard deviation. ^*^ *p* < .05, ^**^ p < .01

Table 1 shows the percentage of categorical demographic variables and the means and standard deviations of the key continuous variables in the hypothesized model. MANOVA was conducted to examine the differences in key variables between heterosexual (*n* = 512), lesbian/gay (*n* = 625), and bisexual (*n* = 183) individuals. The results indicated that there were significant differences in perceived parental (F(2, 1417) = 10.70, *p* < 0.001, partial η^2^ = 0.016) and social pressure to get married (F(2, 1417) = 8.75, *p* < 0.001, partial η^2^ = 0.013), perceived burdensomeness (F(2, 1417) = 40.67, *p* < 0.001, partial η^2^ = 0.058), thwarted belongingness (F(2, 1417) = 33.75, *p* < 0.001, partial η^2^ = 0.049), and suicidality (F(2, 1417) = 52.59, *p* < 0.001, partial η^2^ = 0.074). Post hoc tests were conducted using the Bonferroni correction, indicating that the gay/lesbian group had higher levels of perceived parental (*M*_diff_ = 0.28, *SD* = 0.06, *p* < 0.001) and social pressure to get married (*M*_diff_ = 0.26, *SD* = 0.06, *p* < 0.001), perceived burdensomeness (*M*_diff_ = 0.65, *SD* = 0.07, *p* < 0.001), thwarted belongingness (*M*_diff_ = 0.57, *SD* = 0.07, *p* < 0.001), suicidality (*M*_diff_ = 0.49, *SD* = 0.05, *p* < 0.001) when compared to heterosexual group. In addition, the bisexual group was found to have higher levels of perceived burdensomeness (*M*_diff_ = 0.54, *SD* = 0.11, *p* < 0.001), thwarted belongingness (*M*_diff_ = 0.41, *SD* = 0.10, *p* < 0.001), and suicidality (*M*_diff_ = 0.46, *SD* = 0.07, *p* < 0.001) than heterosexual group. However, there was no significant difference in any variable between lesbian/gay and bisexual group.

In addition, MANOVA and post hoc tests were also conducted to investigate whether there would be differences in suicidality across other demographic variables. The results indicated that individuals who identified with non-binary gender had higher prevalence of suicidality than their peers who identified as male or female, and there was no significant difference between male and female participants. Participants with master or doctorate degree had lower prevalence of suicidality than their peers who received high school education or below, associate degree, or bachelor’s degree. However, there were no significant difference in suicidality between participants who had master and doctorate degree, as well as between participants who received high school education or below, associate degree, and bachelor’s degree. Regarding monthly income, participants whose monthly income ranged from 10,000 to 19,999 RMB or greater than 20,000 RMB had lower prevalence of suicidality than their peers whose monthly income were less than 2,000 RMB, 2,000–4,999 RMB, and 5,000–9,999 RMB per month. However, there were no significant difference in suicidality between participants whose monthly income were 10,000–19,999 RMB or more than 20,000 RMB, as well as between participants whose monthly income were 2,000 RMB, 2,000–4,999 RMB, and 5,000–9,999 RMB. Regarding place of birth, participants who were born in rural areas had lower levels of suicidality than their peers who were born in urban areas. Based on these findings, the variables of gender, educational attainment and monthly income were respectively collapsed into two binary variables: male/female and non-binary gender, bachelor’s degree or below and master degree or above, as well as less than 9999 RMB and more than 9999 RMB per month. The demographic variables of age, gender, educational attainment, monthly income, and place of birth were put as covariates into subsequent analyses.

### Test of the Measurement Model

CFA was conducted to examine the fit of the measurement model with six latent variables: perceived parental, social, and internalized pressure to get married, perceived burdensomeness, thwarted belongingness, and suicidality. All items in the Perceived Parental, Social, and Internalized Pressure to Get Married subscales, Perceived Burdensomeness and Thwarted Belongingness subscales, and the SBQ-R were used as indicators for the corresponding latent variables. Results indicated that the measurement model had an adequate fit to the data (CFI = 0.946; TLI = 0.939; SRMR = 0.058; RMSEA = 0.051, 90% CI [0.049, 0.053]). All factor loadings of the latent variables ranged from 0.54 to 0.93, suggesting that these latent variables were adequately measured by these indicators.

### Test of the Structural Model

We first examined the direct model with sexual orientation status as a predictor, suicidality as an outcome, and age, gender, education, monthly income, and place of birth as covariates. Results suggested that this model had a satisfactory model fit to the data (CFI = 0.972; TLI = 0.958; SRMR = 0.022; RMSEA = 0.050, 90% CI [0.038, 0.060]). Being a sexual minority was associated with higher levels of suicidality (*β* = − 0.25, *p* < 0.001). The direct model explained 14% of the variance in suicidality.

**Fig. 2:**
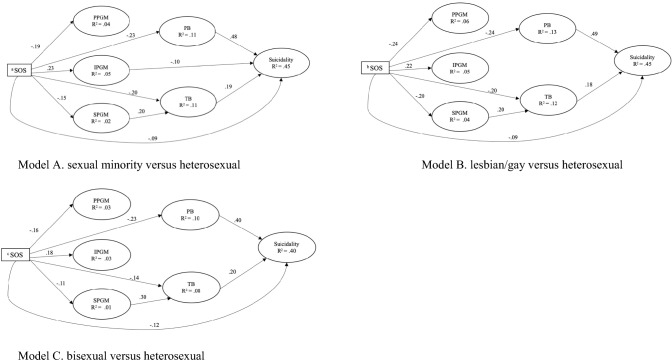
Results of the hypothesized indirect model. *Note*. The significant pathways were displayed in the diagram. PB = Perceived burdensomeness. TB = Thwarted belongingness. SOS = Sexual orientation status. PPGM = Parental pressure to get married. SPGM = Social pressure to get married. IPGM = Internalized pressure to get married. ^a^ SOS was dummy coded, 0 = sexual minority, 1 = heterosexual. ^b^ SOS was dummy coded, 0 = lesbian/gay, 1 = heterosexual. ^c^ SOS was dummy coded, 0 = bisexual, 1 = heterosexual

The hypothesized indirect association between sexual orientation status and suicidality through perceived parental and social pressure to get married and then perceived burdensomeness and thwarted belongingness was examined among all participants. As shown in Table 3, results indicated that this model had an adequate fit to the data. As shown in Table 4 and Fig. 2A, the direct association between sexual orientation status and suicidality became smaller and still significant (*β* = − 0.09, 95% CI [− 0.15, − 0.05]). Four indirect pathways from sexual orientation status to suicidality were identified. The first and second pathways for the indirect association between sexual orientation status and suicidality were respectively through perceived burdensomeness (*β* = − 0.11, 95% CI [− 0.139, − 0.083]) and thwarted belongingness (*β* = − 0.038, 95% CI [− 0.058, − 0.023]), which accounted for 38.87% and 13.43% of the total effect, respectively. Specifically, being a sexual minority was associated with higher levels of perceived burdensomeness (*β* = − 0.23, 95% CI [− 0.28, − 0.18]) and thwarted belongingness (*β* = − 0.20, 95% CI [− 0.25, − 0.13]), which were in turn associated with suicidality (perceived burdensomeness: *β* = 0.48, 95% CI [0.41, 0.55]; thwarted belongingness: *β* = 0.19, 95% CI [0.13, 0.26]). The third pathway for the indirect link between sexual orientation status and suicidality was through internalized pressure to get married (*β* = − 0.024, 95% CI [− 0.044, − 0.005]), which accounted for 8.48% of the total effect. Specifically, being a sexual minority was associated with lower levels of internalized pressure to get married (*β* = 0.23, 95% CI [0.17, 0.29]), which was linked with higher levels of suicidality (*β* = − 0.10, 95% CI [− 0.18, − 0.02]). The fourth pathway for the indirect association between sexual orientation status and suicidality was through perceived social pressure to get married and then thwarted belongingness (*β* = − 0.006, 95% CI [− 0.014, − 0.001]), which accounted for 2.12% of the total effect. Specifically, being a sexual minority was associated with higher levels of perceived social pressure to get married (*β* = − 0.15, 95% CI [− 0.20, − 0.09]), which was linked with higher levels of thwarted belongingness (*β* = 0.20, 95% CI [0.02, 0.36]) and then suicidality. This indirect model explained 45% of the variance in suicidality.

**Table 3:** The model fit indices for the hypothesized indirect models

Model	TLI	CFI	SRMR	RMSEA	90% CI of RMSEA
Model A: sexual minority versus heterosexual	.920	.928	.057	.051	[.049, .053]
Model B: lesbian/gay versus heterosexual	.915	.924	.054	.052	[.050, .055]
Model C: bisexual versus heterosexual	.915	.923	.055	.050	[.047, .054]

TLI = Tucker- Lewis index. CFI = comparative fit index. SRMR = standardized root mean square residual. RMSEA = root mean square error of approximation

**Table 4:** The standardized parameter estimates for the hypothesized indirect models

Parameter estimates	*β*	*SE*	95% CI
Model A: sexual minority versus heterosexual			
^ a^SOS → PPGM	− .19	.03	[− .25, − .14]
^ a^SOS → SPGM	− .15	.03	[− .20, − .09]
^ a^SOS → IPGM	.23	.03	[.17, .29]
^ a^SOS → PB	− .23	.03	[− .28, − .18]
^ a^SOS → TB	− .20	.03	[− .25, − .13]
^ a^SOS → Suicidality	− .09	.03	[− .15, − .05]
PPGM → PB	.08	.06	[− .04, .21]
PPGM → TB	.03	.07	[− .11, .16]
PPGM → Suicidality	.02	.05	[− .07, .13]
SPGM → PB	.12	.08	[− .05, .25]
SPGM → TB	.20	.09	[.02, .36]
SPGM → Suicidality	.01	.06	[− .12, .13]
IPGM → PB	.05	.05	[− .05, .15]
IPGM → TB	.02	.05	[− .09, .12]
IPGM → Suicidality	− .10	.04	[− .18, − .02]
PB → Suicidality	.48	.03	[.41, .55]
TB → Suicidality	.19	.03	[.13, .26]
Age → Suicidality	− .04	.03	[− .09, .01]
^ d^Gender → Suicidality	.08	.03	[.02, .13]
^ e^Educational attainment → Suicidality	− .03	.03	[− .07, .03]
^ f^Monthly income → Suicidality	− .03	.03	[− .07, .02]
^ g^Place of birth → Suicidality	− .03	.02	[− .08, .02]
^ a^SOS → PPGM → Suicidality	− .004	.01	[− .025, .015]
^a^SOS → SPGM → Suicidality	− .001	.009	[− .02, .017]
^ a^SOS → IPGM → Suicidality	− .024	.01	[− .044, − .005]
^ a^SOS → PB → Suicidality	− .11	.014	[− .139, − .083]
^ a^SOS → TB → Suicidality	− .038	.009	[− .058, − .023]
^ a^SOS → PPGM → PB → Suicidality	− .007	.006	[− .022, .002]
^ a^SOS → PPGM → TB → Suicidality	− .001	.003	[− .007, .004]
^ a^SOS → SPGM → PB → Suicidality	− .008	.006	[− .021, .002]
^ a^SOS → SPGM → TB → Suicidality	− .006	.003	[− .014, − .001]
^ a^SOS → IPGM → PB → Suicidality	.005	.006	[− .005, .018]
^ a^SOS → IPGM → TB → Suicidality	.001	.002	[− .004, .006]
Model B: lesbian/gay versus heterosexual			
^ b^SOS → PPGM	− .24	.03	[− .30, − .18]
^b^SOS → SPGM	− .20	.03	[− .25, − .13]
^ b^SOS → IPGM	.22	.03	[.16, .30]
^ b^SOS → PB	− .24	.03	[− .30, − .18]
^ b^SOS → TB	− .20	.04	[− .27, − .13]
^ b^SOS → Suicidality	− .09	.03	[− .16, − .03]
PPGM → PB	.11	.08	[− .05, .25]
PPGM → TB	.03	.09	[− .15, .21]
PPGM → Suicidality	.04	.07	[− .08, .17]
SPGM → PB	.08	.10	[− .11, .27]
SPGM → TB	.20	.11	[− .003, .42]
SPGM → Suicidality	− .01	.08	[− .18, .14]
IPGM → PB	.08	.06	[− .03, .21]
IPGM → TB	.04	.06	[− .07, .16]
IPGM → Suicidality	− .07	.05	[− .16, .03]
PB → Suicidality	.49	.04	[.41, .58]
TB → Suicidality	.18	.04	[.11, .26]
Age → Suicidality	− .05	.03	[− .11, .003]
^ d^Gender → Suicidality	.08	.03	[.02, .15]
^ e^Educational attainment → Suicidality	− .02	.03	[− .07, .04]
^ f^Monthly income → Suicidality	− .02	.03	[− .08, .04]
^ g^Place of birth → Suicidality	− .03	.03	[− .10, .03]
^ b^SOS → PPGM → Suicidality	− .01	.016	[− .044, .018]
^ b^SOS → SPGM → Suicidality	.002	.016	[− .028, .036]
^ b^SOS → IPGM → Suicidality	− .016	.011	[− .038, .006]
^ b^SOS → PB → Suicidality	− .120	.018	[− .158, − .088]
^ b^SOS → TB → Suicidality	− .036	.010	[− .06, − .02]
^ b^SOS → PPGM → PB → Suicidality	− .013	.009	[− .032, .005]
^ b^SOS → PPGM → TB → Suicidality	− .001	.004	[− .011, .006]
^ b^SOS → SPGM → PB → Suicidality	− .008	.010	[− .029, .010]
^ b^SOS → SPGM → TB → Suicidality	− .007	.005	[− .020, − .001]
^ b^SOS → IPGM → PB → Suicidality	.009	.006	[− .003, .023]
^ b^SOS → IPGM → TB → Suicidality	.002	.003	[− .003, .008]
Model C: bisexual versus heterosexual			
^ c^SOS → PPGM	− .16	.04	[− .24, − .08]
^ c^SOS → SPGM	− .11	.04	[− .18, − .02]
^ c^SOS → IPGM	.18	.04	[.09, .26]
^ c^SOS → PB	− .23	.05	[− .32, − .15]
^ c^SOS → TB	− .14	.05	[− .22, − .04]
^ c^SOS → Suicidality	− .12	.04	[− .21, − .04]
PPGM → PB	.04	.08	[− .11, .20]
PPGM → TB	− .05	.09	[− .23, .12]
PPGM → Suicidality	− .01	.07	[− .15, .12]
SPGM → PB	.15	.11	[− .05, .36]
SPGM → TB	.30	.11	[.09, .54]
SPGM → Suicidality	.11	.09	[− .08, .28]
IPGM → PB	− .01	.07	[− .15, .11]
IPGM → TB	− .05	.08	[− .21, .10]
IPGM → Suicidality	− .03	.07	[− .16, .09]
PB → Suicidality	.40	.05	[.30, .51]
TB → Suicidality	.20	.05	[.10, .31]
Age → Suicidality	.05	.04	[− .02, .13]
^ d^Gender → Suicidality	.10	.05	[.01, .19]
^ e^Educational attainment → Suicidality	− .03	.04	[− .11, .05]
^ f^Monthly income → Suicidality	− .06	.04	[− .14, .01]
^ g^Place of birth → Suicidality	− .06	.04	[− .13, .01]
^ c^SOS → PPGM → Suicidality	.002	.011	[− .028, .031]
^ c^SOS → SPGM → Suicidality	− .011	.011	[− .055, .006]
^ c^SOS → IPGM → Suicidality	− .006	.012	[− .047, .022]
^ c^SOS → PB → Suicidality	− .092	.023	[− .195, − .072]
^ c^SOS → TB → Suicidality	− .027	.012	[− .077, − .011]
^ c^SOS → PPGM → PB → Suicidality	− .003	.005	[− .019, .009]
^ c^SOS → PPGM → TB → Suicidality	.002	.003	[− .004, .012]
^ c^SOS → SPGM → PB → Suicidality	− .006	.005	[− .027, .001]
^ c^SOS → SPGM → TB → Suicidality	− .006	.004	[− .023, − .002]
^ c^SOS → IPGM → PB → Suicidality	− .001	.005	[− .015, .011]
^ c^SOS → IPGM → TB → Suicidality	− .002	.003	[− .014, .004]

PB = Perceived burdensomeness. TB = Thwarted belongingness. SOS = Sexual orientation status. PPGM = Parental pressure to get married. SPGM = Social pressure to get married. IPGM = Internalized pressure to get married. ^a^ SOS was dummy coded, 0 = sexual minority, 1 = heterosexual. ^b^ SOS was dummy coded, 0 = lesbian/gay, 1 = heterosexual. ^c^ SOS was dummy coded, 0 = bisexual, 1 = heterosexual. ^d^ Gender was dummy coded, 0 = male or female, 1 = non-binary. ^e^ Educational attainment was dummy coded, 0 = bachelor’s degree or below, 1 = mater degree or above. ^f^ Monthly income was dummy coded, 0 = less than 9999 RMB per month, 1 = more than 9999 RMB per month. ^g^ Place of birth was dummy coded, 0 = urban area, 1 = rural area

To investigate whether the results would be different when comparing specific types of sexual minority groups to heterosexual group, this study further examined the hypothesized indirect model in two subsamples (i.e., a mixed lesbian/gay and heterosexual subsample, a mixed bisexual and heterosexual subsample) after controlling for the same demographic variables. As illustrated in Table 4, the hypothesized model in two subsamples demonstrated adequate fit to the data. In these two models, three indirect pathways from sexual orientation status to suicidality were identified (Please see Table 4, Fig. 2B and 2C). Specifically, sexual orientation status was indirectly associated with suicidality through perceived burdensomeness (a mixed lesbian/gay and heterosexual subsample: *β* = − 0.12, 95% CI [− 0.158, − 0.088]; a mixed bisexual and heterosexual subsample: *β* = − 0.092, 95% CI [− 0.195, − 0.072]) and thwarted belongingness (a mixed lesbian/gay and heterosexual subsample: *β* = − 0.036, 95% CI [− 0.06, − 0.02]; a mixed bisexual and heterosexual subsample: *β* = − 0.027, 95% CI [− 0.077, − 0.011]). The indirect association between sexual orientation status and suicidality was through perceived social pressure to get married and then thwarted belongingness (a mixed lesbian/gay and heterosexual subsample: *β* = − 0.007, 95% CI [− 0.02, − 0.001]; a mixed bisexual and heterosexual subsample: *β* = − 0.006, 95% CI [− 0.023, − 0.002]). These two indirect models respectively explained 45% and 40% for the variance of suicidality.

## Discussion

This is the first study to investigate sexual orientation disparity in suicidality through three types of perceived pressure to get married and then perceived burdensomeness and thwarted belongingness. Within a heteronormative culture characterized by widespread sexual stigma (Liu, 2019), sexual minority adults in the present study reported having higher levels of perceived parental and social pressure to get married, perceived burdensomeness, thwarted belongingness, and suicidality than their heterosexual counterparts. Conversely, they reported lower levels of internalized pressure to get married. Furthermore, four indirect pathways were identified. Among the overall participant sample and the two subsamples, being a sexual minority was indirectly associated with higher levels of suicidality through perceived burdensomeness and thwarted belongingness, as well as through perceived social pressure to get married and then thwarted belongingness. In addition, within the overall participant sample, the indirect association between being a sexual minority and higher levels of suicidality was mediated through internalized pressure to get married.

As expected, sexual minority individuals reported higher levels of perceived parental and social pressure to get married, alongside lower levels of internalized pressure to get married than their heterosexual peers in the overall participant sample. This finding contextualizes previous research that mainly focused on sexual minorities’ experiences with external pressure to get married (Ren et al., 2019; Zheng et al., 2020). In cultures that prioritize heteronormativity and filial piety, heterosexual marriages are not only seen as a developmental milestone but also as a way to honor one’s parents and ancestors (Bedford & Yeh, 2021; Ning & Poon, 2021). While it is reasonable that sexual minorities internalize heteronormative values to a lesser degree compared to their heterosexual counterparts, they have to navigate added minority stress, including sexual orientation identity presentation and management, while public awareness and recognition of diverse intimate partnerships are still evolving. Hence, perceived parental and social pressure to get married may not only persist but also intensify for sexual minorities.

Being a sexual minority was directly associated with higher levels of perceived burdensomeness and thwarted belongingness, supporting our first hypothesis. Sexual orientation disparity in perceived burdensomeness uncovered in the present study is similar to the findings in previous U.S. studies (Hill & Pettit, 2012; Pate & Anestis, 2020). This finding suggests that sexual minorities are more likely to think that they are a burden on significant others across cultures. A higher level of perceived burdensomeness in sexual minorities could be attributed to sexual minority stress (e.g., sexual prejudice and microaggressions), which has received empirical support (Baams et al., 2015). Unlike previous studies that found no significant difference in thwarted belongingness between heterosexual and sexual minority college students in the U.S. (Hill & Pettit, 2012; Pate & Anestis, 2020), this study found that unmarried sexual minority adults endorsed a higher level of thwarted belongingness as compared to their heterosexual peers. The present finding supports the conclusion that sexual minorities are likely to have higher levels of social isolation and loneliness (Hatzenbuehler et al., 2012; Przedworski et al., 2015). In less inclusive environments for sexual minority individuals, sexual minorities are more likely to conceal their sexual orientation and distance themselves from others to protect against discrimination (Pachankis & Bränström, 2018; Wang, 2011).

The second hypothesis received partial support through the identification of four specific pathways linking sexual minority status to suicidality. Based on effect size, the first and second pathways—connecting sexual orientation status to suicidality via perceived burdensomeness and thwarted belongingness—were found to be more prominent than the latter two indirect pathways. These findings align with previous research indicating that perceived burdensomeness and thwarted belongingness contribute to a heightened risk of suicidality within the sexual minority population (Hill & Pettit, 2012; Liu et al., 2023b; Pate & Anestis, 2020). In comparison to the indirect pathway from sexual orientation status to suicidality through thwarted belongingness, the effect size of the pathway through perceived burdensomeness was greater. This observation supports earlier findings that perceived burdensomeness serves as a stronger correlate of suicidality than thwarted belongingness (Chu et al., 2017; Moody et al., 2023). Notably, these two indirect pathways were consistently observed across all participants and within both subsamples, indicating that sexual minorities experience an elevated risk of suicidality through perceived burdensomeness and thwarted belongingness, irrespective of their specific sexual orientation, when compared to their heterosexual counterparts.

The third indirect pathway linking sexual orientation status to suicidality was identified as occurring through internalized pressure to get married in the overall participant sample. This novel finding indicates that sexual minority individuals are less likely to internalize pressure to get married, which then connects with lower risks of suicidality. It appears that internalized pressure to get married may play a protective role in mitigating suicidality. A plausible explanation for this observation is that internalized pressure to get married reflects individuals’ alignment with traditional social norms surrounding marriage, potentially facilitating a sense of harmony between personal beliefs and societal expectations regarding heterosexual marriage. This alignment may further diminish the risk of suicidality (Schneider, 2012). However, this indirect effect did not emerge within either of the two subsamples, suggesting that the effect may be unstable. A possible reason for the lack of significant effects within the two subsamples could be attributed to sample size, as smaller samples are less likely to detect statistically significant relationships (Gannon et al., 2019).

The fourth pathway from sexual minority status to suicidality was identified as occurring through perceived social pressure to get married and then thwarted belongingness. Notably, this fourth pathway, along with the first two pathways demonstrated significance across the overall participant sample and the two subsamples, whereas the third pathway was significant only within the overall participant sample. This indicates that the first, second, and fourth pathways are more likely to co-occur than the third pathway. In addition, the present finding regarding the fourth pathway is consistent with prior research indicating that sexual minorities have an elevated rate of suicidality (Marshal et al., 2011) and that sexual minority stressors are positively associated with suicidality through thwarted belongingness (Liu et al., 2023b). However, the magnitude of this effect was the smallest among the four pathways. One possible explanation is that perceived social pressure to get married is not a stressor uniquely affecting sexual minority individuals, which may account for the weak association between sexual orientation status and perceived social pressure to get married. Furthermore, an increasing number of individuals in China postpone their first marriage or choose to remain unmarried (Yu & Xie, 2021; Zhang et al., 2024), which may diminish the relationship between perceived pressure to get married and thwarted belongingness. Nevertheless, this finding is important, as it offers a novel perspective for understanding the disparity in suicidality between sexual minority and heterosexual groups.

However, this study did not identify the indirect role of perceived burdensomeness in the pathway from sexual orientation status to suicidality through perceived social pressure to get married. The absence of this indirect effect could be attributed to the absence of a significant association between perceived social pressure to get married and perceived burdensomeness. When unmarried adults experience high levels of social pressure to get married, they may interpret this stress as indicative of their deviation from cultural and societal norms (Eklund, 2021), rather than as an affront or a devaluation of their self-worth. Their nonconformity to these cultural and societal norms may hinder them from developing collective identity and fulfilling their need for social belonging (Bedford & Yeh, 2021), which is more closely associated with thwarted belongingness than with perceived burdensomeness. This perspective may elucidate the non-significant association between perceptions of social pressure to get married and perceived burdensomeness.

Moreover, this study did not find any significant connections between perceived parental or internalized pressure to get married with perceived burdensomeness and thwarted belongingness. When parents encourage their children to get married, individuals may not interpret these attitudes and behaviors as signs of parental rejection or animosity; rather, they may view them as expressions of parental care and love (Gui, 2023). This perspective may explain the lack of significant relationships between the variables. Furthermore, internalization of pressure to get married may indicate an alignment with traditional social norms regarding marriage (Zheng et al., 2020). This alignment may serve as a strategy for adapting to societal expectations. Consequently, internalized pressure may not necessarily be associated with perceived burdensomeness and thwarted belongingness.

### Limitations and Future Directions

This study enriches our understanding of how sexual orientation status contributes to suicidality, albeit with limitations worth attention. First, this study used a non-probability sampling method, resulting in a sample characterized by a higher proportion of females, younger individuals, and participants with greater educational attainment, as well as a tendency to have been born in urban areas compared to the general population. Researchers are recommended to use probability sampling methods to obtain a more representative sample. Second, given the limited number of queer, questioning, pansexual, and asexual individuals, these identities were grouped with other sexual minority identities in comparison to heterosexual individuals in the hypothesized indirect path model rather than analyzing them as standalone identities. Researchers may consider recruiting larger samples of individuals with these identities to examine potential differences in mental health needs across subgroups of sexual minorities. Third, this study employed a cross-sectional design, limiting the ability to draw causal inferences. Future studies should consider using longitudinal designs to examine the temporal and directional relationships between variables in the hypothesized model.

### Implications

Despite these limitations, the present findings have significant implications for practice and theory. The four identified indirect pathways are significant as they offer insights into strategies for reducing the elevated risks of suicidality among unmarried sexual minority individuals. Specifically, suicidal intervention programs may benefit from acknowledging the contribution of sexual orientation minority status to internalized and perceived social marriage pressure, perceived burdensomeness, and thwarted belongingness. Informed by the serial mediation link via perceived social pressure and thwarted belongingness, mental health professionals may support their Chinese sexual minority clients at risk of suicide by facilitating discussions on how social expectations of entering a heterosexual marriage impact their mental health in the context of their sexual minority orientation. Therapists may integrate cognitive behavioral therapy into sexual minority affirmative practice to address negative self-perceptions arising from such expectations (Dozois & Beck, 2023; Pachankis et al., 2015). Incorporating mindfulness and compassion-based psychotherapy may also assist sexual minority clients in managing the feelings of thwarted belongingness (Bianchini & Bodell, 2024).

From a policy perspective, the legal recognition of same-sex relationships could help further destigmatize and normalize sexual minority orientations, thereby reducing perceived social pressure to enter a heterosexual marriage among sexual minorities. This policy change may also support sexual minorities in fostering a sense of belongingness within broader society even if social pressures to get married do not dissipate immediately. A reduced sense of thwarted belongingness could subsequently alleviate suicidality among sexual minorities. From a theoretical perspective, this study identified significant disparities in perceived parental, social, and internalized pressure to get married, as well as perceived burdensomeness, thwarted belongingness, and suicidality between unmarried sexual minority adults and their heterosexual counterparts. Moreover, it elucidated possible pathways linking sexual orientation status to suicidality.

## Data Availability

The datasets and material in the current study are available from the corresponding author upon reasonable request.

## Code Availability

The code in the current study is available from the corresponding author upon reasonable request.
